# Exploring the relationship between muscle activity, jaw behaviour and pain

**DOI:** 10.1038/s41598-025-22184-y

**Published:** 2025-10-07

**Authors:** Nikola Stanisic, Sara Baram, Laura Nykänen, Thomas List, Alessandro Bracci, Peter Svensson, Daniele Manfredini, Birgitta Häggman-Henrikson

**Affiliations:** 1 Department of Orofacial Pain and Jaw Function, Faculty of Odontology, Malmö University, 205 06 Malmö, Sweden; 2 Section for Orofacial Pain and Jaw Function, Institute for Odontology and Oral Health, Aarhus University, Aarhus, Denmark; 3 Department of Odontology, Faculty of Health and Medical Sciences, University of Copenhagen, Copenhagen, Denmark; 4 Clinic of Oral and Maxillofacial Diseases, Helsinki University Hospital, Helsinki, Finland; 5 Department of Oral and Maxillofacial Diseases, University of Helsinki, Helsinki, Finland; 6 Department of Rehabilitation Medicine, Skåne University Hospital, Lund, Sweden; 7 School of Dentistry, Department of Neurosciences, University of Padova, Padua, Italy; 8 Faculty of Dentistry, National University of Singapore, Singapore, Singapore; 9 Department of Medical Biotechnologies, University of Siena, Siena, Italy

**Keywords:** Awake bruxism, Ecological momentary assessment, Electromyography, Muscle overload, Temporomandibular disorders, Health care, Medical research, Risk factors

## Abstract

While muscle overload is commonly implicated in musculoskeletal pain conditions, real-time assessment of associated behavioural and physiological features is challenging. This study aims to investigate the relationship between self-reported awake bruxism using Ecological Momentary Assessment (EMA) and jaw muscle activity registered by surface electromyography (sEMG), and differences between individuals with and without temporomandibular disorder (TMD) pain. Seventy participants (38 women, 32 men), of which 31% reported pain, completed 3-day EMA using a smartphone application combined with a sEMG device only for day 1. Overload, defined as muscle activity exceeding 20% of maximum voluntary contraction (MVC), was evaluated for duration and area under curve (AUC). A strong correlation was observed between EMA-reported bruxism and sEMG overload duration (ρ = 0.62, *p* < 0.001). AUC showed a correlation with EMA only in the TMD group. Participants with TMD pain exhibited shorter high-intensity bursts (60–79% MVC, *p* ≤ 0.005) but prolonged low-intensity muscle activity (20–39% MVC, *p* < 0.001). Bruxism behaviour and stress levels were higher in women and in individuals with pain. The results suggest that combining EMA and sEMG provides valid assessment of musculoskeletal overload, capturing both perceptual and physiological dimensions. Incorporating EMA in pain management can identify pain-related risk behaviours, thus supporting tailored patient-centred interventions.

## Introduction

Musculoskeletal overload refers to a state in which muscles are subjected to mechanical or functional short- or long-term demands that may exceed their capacity to recover^1,2^. This relationship between functional capacity and load of the musculoskeletal system is dynamic and varies between individuals, depending on physiological, psychological, and contextual factors. Under normal conditions, the balance between load and capacity enables adequate function and recovery^3,4^. However, when the load consistently exceeds the capacity of the musculoskeletal system and its ability to recover, a cumulative strain may develop, leading to pain and alterations in motor output and motor control^5,6^. It has been proposed that this may initiate a vicious cycle where pain itself contributes to further motor adaptations, reinforcing overload, and thereby maintaining the pain state. In contrast, the pain adaptation model predicts pain-related changes in motor behaviour. While these can initially be protective, they may affect motor variability and redistribute load in a way that increases sustained muscular activity^6,7^. This pathophysiological process is especially relevant in common musculoskeletal conditions such as neck and back pain^8^, as well as pain in the orofacial region related to temporomandibular disorders (TMDs)^9,10^.

Despite the clinical relevance of musculoskeletal overload, it is challenging to assess both in the clinic and in research settings due to its fluctuating and often subconscious nature^11,12^. Conventional methods for assessment, such as questionnaires or interviews, are often based on single-point measures and limited by recall bias. Ecological momentary assessment (EMA) has been proposed as a more valid alternative in pain research and management, as it captures data in real time and within natural contexts with the added benefit of providing longitudinal data^13^. By using repeated self-report via smartphone apps, EMA can provide longitudinal, multi-point measures with reduced recall bias^14,15^. This also allows for tracking day-to-day variability in behaviours and symptoms, offering more ecologically valid insights into musculoskeletal pain and behaviour^16–18^.

Psychosocial factors, especially stress, can influence muscle activity, and importantly, the perception of muscle activity and thereby contribute to musculoskeletal overload^19^. One notable example is awake bruxism (AB)—a group of mostly subconscious, repetitive non-functional jaw muscle activities such as clenching, grinding, or bracing—affecting approximately 25% of the general population^20,21^. These repetitive non-functional activities can place prolonged mechanical stress on the jaw muscles and joints, which may be particularly relevant in TMD pain^22,23^. Given that awake bruxism is often a stress-related behaviour and that long-term stress has been linked to central sensitization, it is reasonable to assume that stress may not only lead to muscle overload but can also contribute to heightened pain sensitivity^24,25^.

TMDs are among the most prevalent chronic pain conditions, affecting approximately 10% of adults^26^, and population-based data suggest that the burden of TMDs is increasing, especially among young adults and women^27^. TMDs also reflect mechanisms observed in other musculoskeletal disorders, such as altered pain modulation and perturbed neuroplasticity^28,29^. The masticatory system, comprising the jaw muscles, the temporomandibular joints, and associated neural structures, offers a compelling and accessible study model for understanding the relationship between stress-related musculoskeletal overload and pain.

Several studies have demonstrated the feasibility and clinical value of EMA for assessing jaw-related behaviours and orofacial pain in real-world settings^22,23,30,31^. However, while EMA captures subjective experiences and behavioural patterns, it does not provide direct physiological measures of muscle activity. Surface electromyography (sEMG) offers a non-invasive, neurophysiological method to quantify muscle activity and has been used to evaluate muscle overload patterns^32^. Combining EMA and portable sEMG provides the opportunity of simultaneously capturing self-reported behaviours and objective measures of such overload. Given the multifactorial nature and rising burden of chronic musculoskeletal pain, assessment methods need to reflect both perceived and physiological aspects of muscle overload. Taken together, combining EMA and EMG may offer a powerful approach to understanding pain-related behaviour and physiological responses in real time.

The primary aim of this study was to investigate the relationship between self-reported awake bruxism behaviours, assessed through EMA, and masticatory muscle activity recorded by portable sEMG. The secondary aim was to compare EMA-reported behaviours and sEMG-recorded muscle activity between individuals with and without TMD pain.

The primary hypothesis was that a higher frequency of self-reported awake bruxism behaviours, as assessed by EMA, is associated with increased masticatory muscle activity. The secondary hypothesis was that individuals with TMD pain exhibit a higher frequency of EMA-reported awake bruxism behaviour and a higher degree of muscle activity compared to individuals without TMD pain.

## Methods

### Participants

Participants were recruited through public advertisements at Malmö University and Skåne University Hospital, Sweden. Inclusion criteria included daily use of a smartphone, fluency in Swedish, and age 18–65 years. Exclusion criteria were factors affecting the attachment of the sEMG device, such as excessive facial hair (e.g., thick beard) or skin conditions hindering electrode adhesion.

### Procedure

Prior to the assessment, participants completed a three-item validated screening tool for TMD (3Q/TMD), together with the DC/TMD Symptom Questionnaire (SQ/TMD)^33,34^, which is used to assess symptoms needed for a clinical diagnosis. Participants also rated average TMD pain intensity using a numeric rating scale (NRS, 0–10) and completed the Perceived Stress Scale (PSS-10). Jaw opening capacity was measured using a millimetre ruler, with participants instructed to open their mouth as wide as possible even if painful.

TMD pain status was defined as positive if participants provided affirmative responses to both the 3Q/TMD and the SQ/TMD, specifically reporting facial pain in the jaw, temple, ear, or in front of the ear during the past 30 days, and indicating that this pain was frequent (once a week or more) and modified by jaw function, such as chewing, talking, or yawning.

Prior to data collection, participants received standardized instructions to ensure they could accurately recognize and differentiate the five jaw muscle conditions associated with awake bruxism^23^. Participants then used a smartphone-based EMA app (BruxApp – WMR srl, World Medical Applications, Italy) to report their awake bruxism activity over a 3-day period, a duration that was chosen to balance ecological validity and participant compliance, which is in line with previous studies demonstrating reliable day-to-day measurement of jaw muscle activity^35^. The smartphone-app generated notifications at random times throughout the day, prompting participants to identify their current jaw position in real-time by selecting one of five conditions: relaxed jaw muscles, light teeth contact, teeth clenching, grinding, or mandible bracing. This method enabled real-time, context-specific self-reporting of awake bruxism activity. Participants were required to respond to at least 12 EMA prompts per day^36^.

**Fig. 1:**
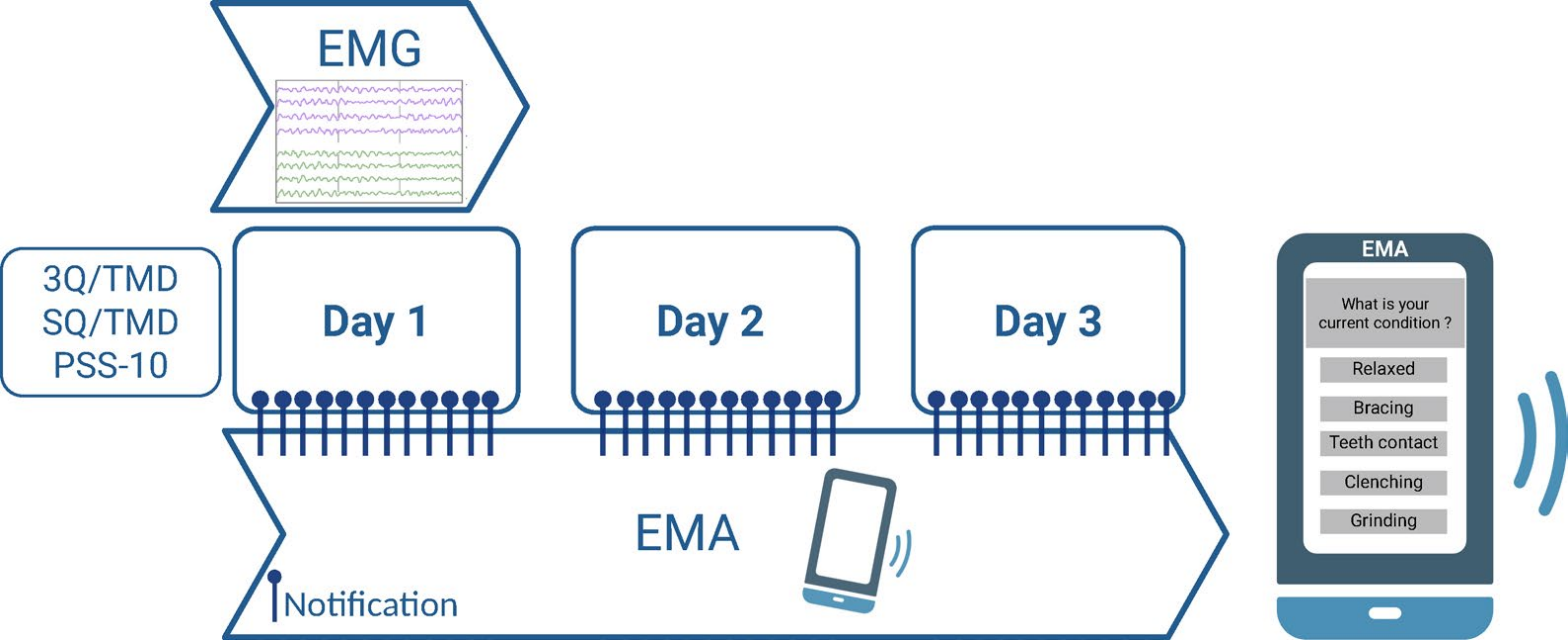
Study protocol combining electromyography (EMG) Day 1 and Ecological Momentary Assessment (EMA) Days 1–3 after baseline assessment of Temporomandibular disorders (TMD) with 3Q/TMD (3-item TMD screening) and SQ/TMD (Symptom Questionnaire for TMD); and assessment of stress with PSS-10 (Perceived Stress Scale-10).

The first day of the EMA period was scheduled on a Monday or a Tuesday to ensure that the participants experienced a typical working weekday. On this first day of registration, jaw muscle activity was recorded simultaneously using a portable sEMG device (dia-BRUXO, Biotech-Novations, Sanremo, Italy). EMG was recorded on Day 1 to provide an objective, validated measure of jaw muscle activity. Because EMA relies on self-report, the EMA protocol was designed with two additional consecutive days. This design ensured both validation of EMA against concurrent EMG on Day 1 and captured day-to-day variation in self-reported AB behaviour. The portable sEMG device was attached to the skin over the bulky part of the left masseter muscle (Fig. 1). The device features a sensitive amplifier that captures low-amplitude EMG signals via disposable bipolar electrodes (Ag/AgCl, solid gel) with a 22 mm fixed inter-electrode distance. Signals were processed through a three-stage analogue circuit comprising amplification, a bandpass filter (110–550 Hz), and a root mean square (RMS) integrator. This raw EMG data was extracted from each device after analogue processing on the same day as the recording to calculate the mean microvolt (µV) value for each millisecond. For final analysis, the data were converted to mean µV per second. Both the sEMG device and EMA application were set up and activated before 09:45 a.m., ensuring data collection throughout both morning and afternoon hours. Participants were requested to wear the device for a minimum of seven continuous hours. Jaw opening capacity was measured before and after sEMG device attachment to ensure the device did not restrict jaw function.

Throughout the sEMG registration period, participants also completed a diary, noting periods of functional activity such as eating or prolonged speaking, specifying the type of activity and corresponding time frames. Based on these diary entries, EMG curves were visually inspected to remove from the analysis time periods corresponding to functional activities such as eating and talking. As a result, only non-functional muscle activity was used in the analysis. All reported diary events from the participants were verified by inspecting the corresponding time stamps in the raw EMG data before exclusion.

### Overload definition

Maximal voluntary contraction (MVC) was assessed at the start of the EMG recording by instructing each participant to perform a voluntary maximal jaw clench. The average sEMG signal from the first 5 s of this contraction was used to establish the individual’s MVC baseline. Masticatory muscle overload was defined as sustained EMG activity exceeding 20% of the individual’s MVC^37–41^.

### Data and statistical analysis

The prevalence of awake bruxism in the general population has been estimated at 25.9% (95% CI 22.2–29.9), based on a recent systematic review and meta-analysis^20^. Using this prevalence, a 95% confidence level and a 10% margin of error, the required sample size was 70 participants.

Descriptive statistics were used to summarize demographic, behavioural, and physiological variables. Continuous variables, including age (years), maximum voluntary contraction (MVC, µV), jaw opening capacity (mm), EMG overload (minutes), awake bruxism frequency on Day 1 and averaged across Days 1–3 (% AB), Perceived Stress Scale scores (PSS-10, range 0–40), and average pain intensity (Numeric Rating Scale, NRS; range 0–10) were reported as means and standard deviations (SD). Categorical variables, such as sex (men/women) and TMD pain status (TMD pain versus No TMD pain) were reported as frequencies and percentage. Group differences between women and men, and between participants with and without TMD pain, were analysed using independent samples t-tests for variables that were normally distributed. The assumption of normality was evaluated using the Shapiro–Wilk test. For categorical variables, such as the distribution of sex across TMD pain and No TMD pain groups, chi-square tests were used.

Different types of self-reported awake bruxism behaviour (teeth contact, clenching, grinding, or bracing) collected via EMA was grouped as a categorical variable and compared to reported relaxed state. In the analysis, these responses were aggregated into proportions, making them continuous variables representing the percentage of self-reported bruxism-related behaviours.

EMG overload was defined and assessed in two ways:

1. Duration-based overload, calculated as the total number of seconds per day during which the EMG signal exceeded 20% of the individual’s MVC, excluding periods of functional activity (e.g., eating or speaking); and
2. Intensity-based overload, measured as the area under the curve (AUC) of the EMG signal above the 20% MVC threshold (in µV s), calculated by integrating the RMS-processed EMG signal (in µV) over time (in seconds), reflecting both duration and magnitude of muscle activity.

Perceived stress was assessed using the PSS-10 questionnaire, which yields a total score from 0 to 40, calculated by summing responses to 10 items, each scored on a 5-point Likert scale (0 = never to 4 = very often). Associations between these continuous measures (EMA proportions, EMG overload in seconds and PSS-10) were evaluated using Spearman’s rank correlation coefficient^42^. Based on established thresholds, the strength of correlations was interpreted as weak (0.20–0.39), moderate (0.40–0.59), or strong (≥ 0.60)^43^. To evaluate within-group changes in EMA-derived bruxism activity across 3 days, the Friedman test was used as a non-parametric alternative to repeated measures ANOVA. For significant Friedman test results, Wilcoxon signed-rank tests were conducted as post hoc pairwise comparisons between the days.

To evaluate within-subject changes in EMA-derived bruxism activity (i.e., percentage of self-reported awake bruxism events) across three consecutive days, the Friedman test was used as a non-parametric alternative to repeated measures ANOVA. This approach accounts for the non-normal distribution and repeated nature of the data. For significant Friedman test results, Wilcoxon signed-rank tests were conducted as post hoc pairwise comparisons between individual days. Statistical analyses were performed using Prism (version 10.2.0 (355)) and illustrations using BioRender. All tests were two-tailed, and significance was set at *p* < 0.05.

### Ethics

The study was approved by the Swedish Ethical Review Authority (Dnr 2022-06841-01/2024-05598-01). All participants provided written informed consent after receiving written and verbal information about the general aim of the study. All methods were performed in accordance with the relevant guidelines and regulations.

## Results

A total of 70 participants (38 women, 32 men) were included in the study. Across the 3-day EMA period, compliance was high, with participants responding to a mean of 15.7 notifications per day, corresponding to 79% of all scheduled notifications. All 70 participants completed the study and reached the minimum threshold of 12 responses/day.

In total, 48 participants (69%) were classified as No TMD pain and 22 (31%) were classified as TMD pain cases. Men had a higher MVC (*p* < 0.001) and jaw opening capacity compared to women (*p* < 0.001). Participants in the No TMD pain group showed higher MVC (*p* < 0.001) and greater jaw opening (*p* < 0.001) compared to individuals with TMD pain (Table 1). The mean NRS score for average pain in the TMD pain group was 5.4 (SD 2.0; range: 2–9) with no significant difference between women and men.

**Table 1:** Participant characteristics and comparison of study variables stratified by sex and TMD pain status.

Variable	Total	Women, n (%)	Men, n (%)	*p*-value	No TMD pain	TMD pain	*p*-value
Participants (n)	70	38 (54%)	32 (46%)		48 (22 ♀)	22 (16 ♀)	0.079^3^
Mean age years (SD)	33 (6)	33 (5)	32 (6)	0.285^1^	33 (6)	32 (6)	0.195^1^
Recording time minutes (SD)	433 (25)	435 (26)	430 (23)	0.334^2^	433 (26)	435 (23)	0.091^2^
Mean MVC µV (SD)	402 (42)	377 (34)	430 (32)	**< 0.001**^**1**^	415 (40)	379 (37)	**< 0.001**^**1**^
Jaw opening mm (SD)	47 (6)	44 (5)	51 (6)	**< 0.001**^**1**^	49 (5)	42 (4)	**< 0.001**^**1**^
EMG overload minutes (SD)	17 (5)	18 (5)	16 (5)	0.120^1^	15 (4)	21 (4)	**< 0.001**^**1**^
EMG overload µV min (SD)	2795.1 (854.2)	2721.8 (687.4)	2919.6 (746.6)	0.264^1^	2692.7 (774.5)	3018.3 (551.1)	0.084^**1**^
EMA day 1: %AB (SD)	16 (7)	18 (7)	13 (6)	**< 0.001**^**1**^	13 (5)	23 (5)	**< 0.001**^**1**^
EMA days 1–3: %AB (SD)	15 (7)	17 (8)	12 (6)	**0.004**^**1**^	11 (5)	22 (5)	**< 0.001**^**1**^
PSS-10 mean (SD)	17 (6)	18 (5)	15 (6)	**0.008**^**1**^	14 (5)	22 (4)	**< 0.001**^**1**^

TMD, Temporomandibular disorder; SD, standard deviation; MVC, maximum voluntary contraction; EMG, electromyography; µV, microvolt; µV s, microvolt-seconds; EMA, ecological momentary assessment; AB, awake bruxism; PSS-10, perceived stress scale, 10-item version.

^1^Independent samples *t*-test, ^2^Mann–Whitney U test, ^3^Chi-square test. Tests were assigned based on the Shapiro–Wilk normality check for continuous variables and applied accordingly to each row. Significant values are in bold.

**Fig. 2:**
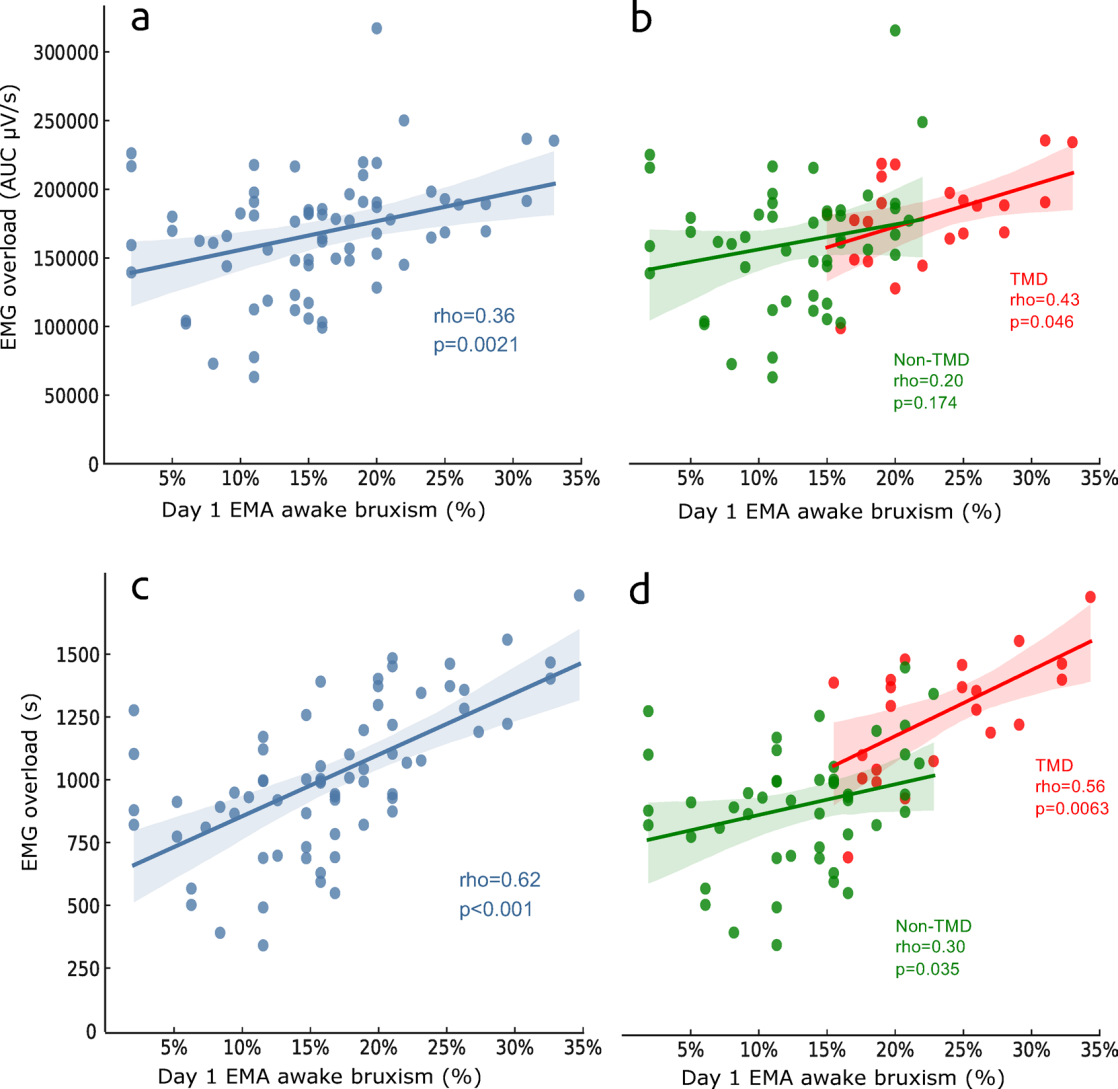
Associations (Spearman’s correlation) between Ecological Momentary Assessment (EMA) of awake bruxism on Day 1 and Electromyography (EMG) of muscle overload assessed by area under curve (AUC) in the total sample (**a**, **c**) and stratified by Temporomandibular disorder (TMD) pain status (**b**, **d**).

Participants with TMD pain had significantly longer EMG overload durations compared to No TMD pain participants (21 min vs. 15 min; *p* < 0.001). There was a strong positive correlation (ρ = 0.62, *p* < 0.001) between EMA-reported awake bruxism and duration of jaw muscle activity above the 20% MVC threshold (Fig. 2). When stratified by group, this correlation was moderate for the TMD pain group (ρ = 0.56, *p* = 0.006) and weak for the No TMD pain group (ρ = 0.30, *p* = 0.035) (Fig. 2), but with no significant difference in the strength of correlation between the two groups (*p* = 0.90).

EMG overload was also quantified using area under the curve (AUC, µV*s) to reflect both the duration and intensity of muscle activity above the 20% MVC threshold. There was no significant difference in AUC between TMD pain and No TMD pain groups (*p* = 0.084). In the total sample, a significant positive correlation was found between EMA-reported awake bruxism and AUC (ρ = 0.36, *p* = 0.002) (Fig. 2). When stratified by group, this correlation was moderate for the TMD pain group (ρ = 0.43, *p* = 0.046), whereas there was no significant correlation for the No TMD pain group (ρ = 0.20, *p* = 0.174) (Fig. 2).

EMG overload duration stratified by MVC intensity levels showed significant group differences in several ranges of overload (Table 2). P-values were Bonferroni-adjusted for eight comparisons (α = 0.05/8 = 0.00625). Participants with TMD pain exhibited significantly shorter high intensity durations in the 60–69% MVC (*p* = 0.005) and 70–79% MVC ranges (*p* < 0.001) but significantly more time in the 20–29% MVC (*p* < 0.001) and 30–39% MVC (*p* < 0.001) ranges compared to the No TMD pain group (Table 2).

**Table 2:** Mean duration (seconds) of EMG overload across different ranges of maximum voluntary contraction (MVC) intensity, for participants with and without Temporomandibular Disorder (TMD) pain.

MVC % range	No TMD pain (n = 48) (s)	TMD pain (n = 22) (s)	*p*-value
20–29	197.7	438.9	**< 0.001**^1^
30–39	211.0	340.5	**< 0.001**^1^
40–49	197.7	226.3	0.02^1^
50–59	135.7	138.6	0.717^1^
60–69	90.4	76.2	**0.005**^1^
70–79	53.8	37.9	**< 0.001**^1^
80–89	5.5	3.8	0.039^2^
90–100	5.1	3.4	0.087^2^

^1^Independent samples *t*-test, ^2^Mann–Whitney U test was applied to each row based on the Shapiro–Wilk normality check.

*p*-values adjusted for multiple comparisons using Bonferroni correction (α = 0.00625). Significant values are in bold.

**Fig. 3:**
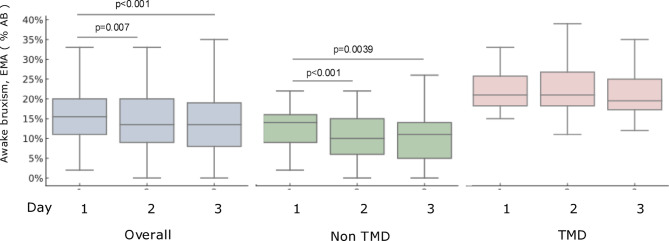
Box and whisker plots (median, quartile, 10% and 90%) of Ecological Momentary Assessment (EMA) reported awake bruxism (%) across three consecutive days, for the whole group and stratified by Temporomandibular Disorder (TMD) pain status.

Reported awake bruxism activity was higher in women than in men both on Day 1 (18% vs. 13%; *p* < 0.001), and when averaged across Days 1–3 (17% vs. 12%; *p* = 0.004) (Table 1). Compared to individuals without TMD pain, participants with TMD pain reported higher activity both on Day 1 (23% vs. 13%; *p* < 0.001) and when averaged across Days 1–3 (22% vs. 11%; *p* < 0.001) (Table 1). The reported awake bruxism activity varied significantly across days in the overall sample (χ^2^ = 13.77, *p* = 0.001). In the No TMD pain group, a similar pattern was observed (χ^2^ = 14.94, *p* = 0.0006). No significant day-to-day variation was found in the TMD group (χ^2^ = 4.67, *p* = 0.097) (Fig. 3).

**Fig. 4:**
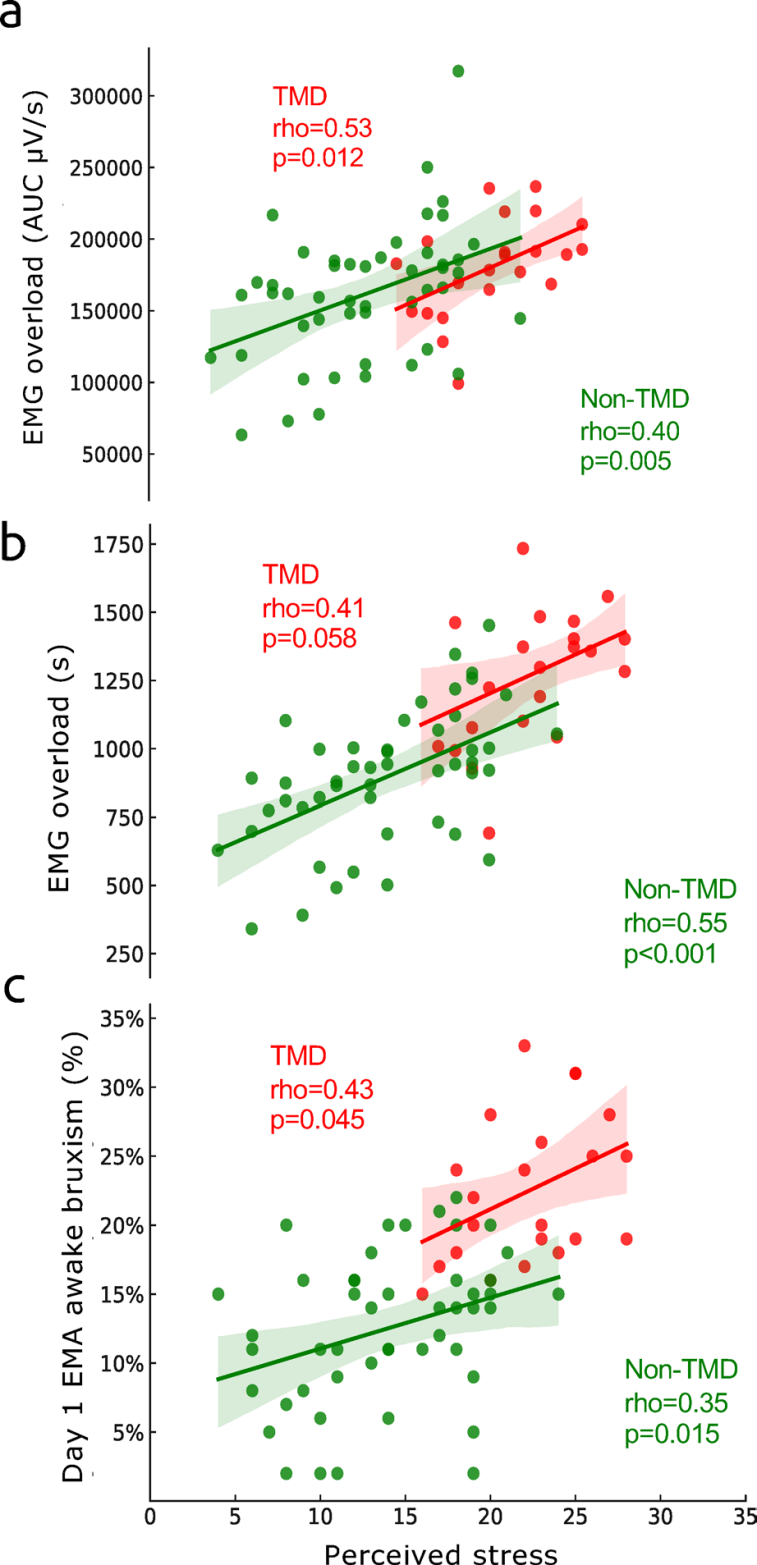
Associations (Spearman’s correlation) between perceived stress and (**a**, **b**) Electromyography (EMG) overload assessed by area under curve (AUC) and time; and (**c**) Ecological Momentary Assessment (EMA) reported awake bruxism on Day 1; stratified by Temporomandibular disorder (TMD) pain status.

Women reported significantly higher PSS-10 scores than men (*p* = 0.008) (Table 1). Participants in the TMD group reported significantly higher PSS-10 scores compared to those in the No TMD pain group (*p* < 0.001) (Fig. 4).

**Fig. 5:**
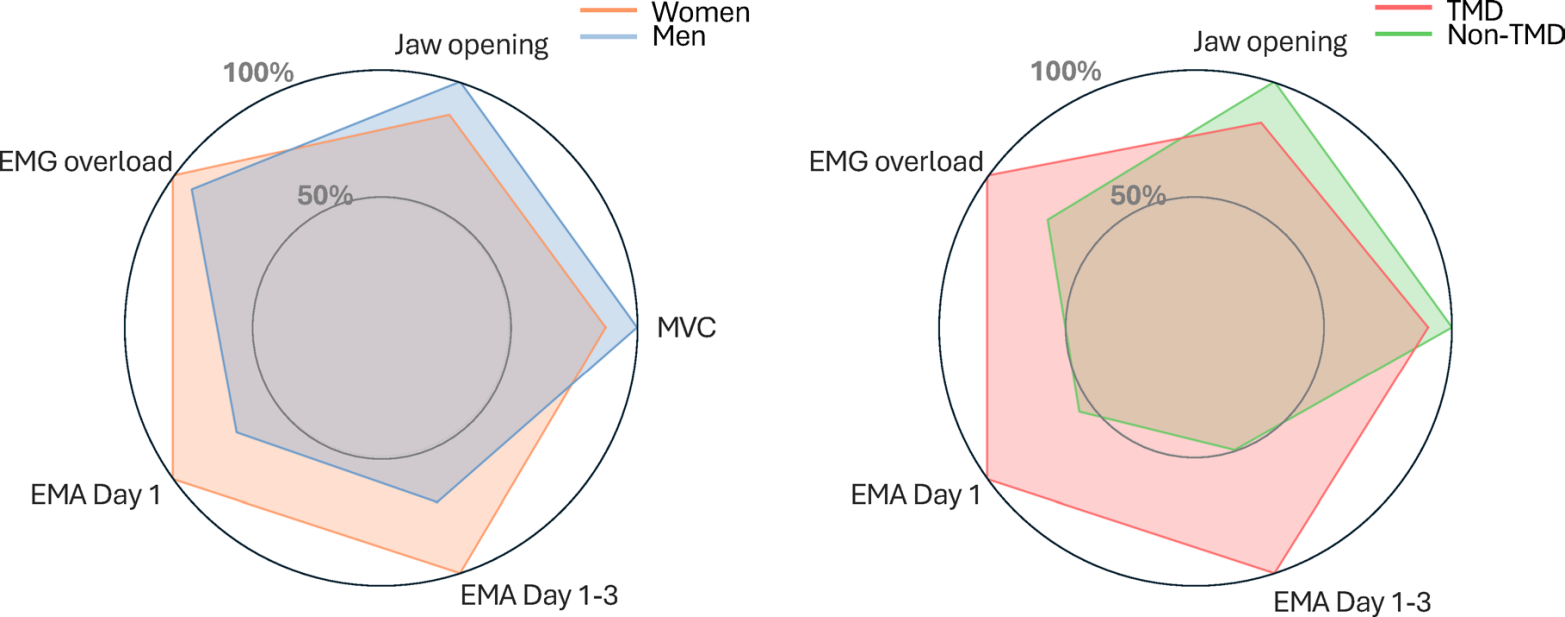
Radar plots comparing normalized functional and non-functional measures by sex (left) and Temporomandibular disorder (TMD) pain status (right). The five axes represent maximum voluntary contraction (MVC), jaw opening capacity, electromyography (EMG) overload duration (minutes), and awake bruxism (AB) reported via Ecological Momentary Assessment (EMA) on Day 1 and averaged across Days 1–3. Values are normalized (0–100%) based on the highest observed value in the total sample. Left plot compares women (orange) and men (blue). Right plot compares individuals with (red) and without (green) TMD pain.

Overall profiles of functional and non-functional variables are presented as radar charts for men versus women and for groups with and without TMD pain (Fig. 5).

## Discussion

The main finding of the present study was a significant association between self-reported awake bruxism behaviours and objectively measured, non-functional muscle activity. This confirms the primary hypothesis of the study and suggests that participants have a degree of momentary awareness of non-functional jaw muscle activity and that EMA can successfully detect clinically relevant patterns of muscle overload. The secondary hypothesis—that individuals with TMD pain exhibit higher frequency of bruxism behaviour and higher degree of muscle activity compared to individuals without TMD pain—was partly confirmed. Individuals with TMD pain self-reported a higher degree of awake bruxism activity and demonstrated longer durations of muscle activity in lower intensity ranges.

TMD pain, a musculoskeletal condition that is the most common origin of chronic orofacial pain, is known to alter the neurophysiological control of the jaw system. Specifically, orofacial pain has been shown to interfere with neuroplasticity in the primary motor cortex, limiting the capacity for sensorimotor adaptation and new motor learning^44,45^. Simultaneously, changes in the primary somatosensory cortex can lead to heightened sensitivity to orofacial input. Orofacial sensory input, specifically from periodontal receptors, has been studied extensively by Trulsson et al., who demonstrated their role in encoding forces and contributing to fine motor control during mastication and other jaw behaviours^46,47^. Orofacial pain has been reported to alter trigeminal sensorimotor function, affecting reflex responses and fine motor coordination, potentially acting as a warning system during functional jaw activities^44,45^. This aligns with findings of higher occlusal tactile acuity, provided by the periodontal receptors, in TMD pain patients, suggesting that pain can amplify local sensory discrimination in the jaw system^48^. It has also been suggested that such changes may reflect a form of heightened alertness or protective tuning in the sensorimotor system, possibly aimed at minimizing further strain or injury^49^. While this mechanism may be adaptive in acute contexts, in chronic pain conditions such as TMD, it could contribute to an exaggerated perception of sensorimotor input related to normal muscle activity. Thus, whereas some studies among patients with TMD pain show impaired tactile resolution in extraoral regions^50,51^, intraoral regions may demonstrate localized sensory amplification related to nociceptive input. This redistribution of sensory attention could increase the awareness of otherwise low-level muscle activity. However, experimental studies show that even strenuous jaw activity in healthy individuals often leads to only mild non-painful symptoms such as fatigue or stiffness, suggesting that overload alone is not always sufficient to produce pain. Such mechanisms may in part explain why, in our study, participants with TMD pain showed a moderate correlation between self-reported awake bruxism and overload as registered by EMG, whereas the No TMD pain group showed only a weak correlation. This finding suggests that the overall association in the total sample was primarily driven by participants with TMD pain.

It is important to consider that awake bruxism is not a single activity but rather a spectrum of activities, including teeth clenching, grinding, and jaw bracing. It was previously reported that individuals who frequently report tooth clenching during the day via EMA also show increased masseter muscle activity on concurrent EMG recordings^39^. However, the study focused specifically on only one aspect of awake bruxism behaviour—tooth clenching—and included only pain-free individuals, limiting generalizability to the full spectrum of awake bruxism behaviours and to clinical populations^39^.

Different awake bruxism behaviours likely differ in both motor demands and perceptual salience. For example, tooth-contact behaviours such as clenching may be easier for the individual to detect than non-contact muscle bracing that does not activate periodontal receptors, particularly in study populations with pain-induced increased somatic vigilance. The mean reported pain intensity in our TMD pain group was 5.4 and thus at the higher end of the 4.3–5.3 NRS range observed in comprehensive studies such as the OPPERA study^52,53^. This suggests that our study sample had a clinically meaningful level of pain and thereby potentially heightened perceptual gain, in line with prior findings of somatosensory amplification and attentional bias in individuals with TMD pain^54^. This may have contributed to the significant correlation between EMA self-report and EMG-measured muscle activity in the TMD pain group. From a clinical perspective, this provides support for the use of EMA in populations with overload behaviour, not only related to jaw function but also in a more general context of musculoskeletal overload.

The TMD pain group showed stable levels of self-reported awake bruxism across the 3-day EMA period, whereas the No TMD pain group exhibited a significant reduction from Day 1 to Day 3. While awake bruxism is typically considered a fluctuating behaviour influenced by a combination of internal and external factors, the decrease in the No TMD pain group may reflect a feedback effect of EMA itself by raised awareness, prompting immediate behavioural change. In contrast, individuals with TMD pain may exhibit more rigid and engrained patterns of jaw muscle activity that are less responsive to self-monitoring and intervention alone. This could reflect an “overtrained” state^55,56^, where repetitive behaviours such as awake bruxism become habitual and less consciously regulated.

This interpretation is supported by research on neuroplasticity. In a systematic review, we showed that pain, both acute and chronic, can disrupt training-induced neuroplasticity, which is an important aspect in motor adaptation^57^. In line with this, Ikuta et al. showed that individuals with bruxism failed to show typical corticomotor plasticity after jaw training tasks^55^. Complementing these findings, Boscato et al. reported that after masseter sensitization, bruxers showed reduced changes in corticomotor excitability compared to non-bruxers^56^. Taken together, these findings indicate that once muscle overload behaviours become firmly established, particularly in the context of pain, such behaviours may shift from being flexible to more rigid. This could help explain why the TMD pain group did not show the same reduction in awake bruxism over time as in the No TMD pain group. Awareness alone may not be sufficient to modify overload behaviours in the presence of pain-related overload. Behavioural interventions in patients with chronic pain conditions may therefore require more structured and multimodal retraining approaches, such as cognitive–behavioural strategies together with the evaluation of possible underlying psychological factors predisposing overload behaviours^25^.

This view of a more rigid and complex system in pain patients is further supported by our data on perceived stress. In the pain-free group, perceived stress was significantly correlated with both EMA-reported overload behaviour and EMG-registered muscle activity, suggesting that the reported overload was stress-related. However, in the TMD pain group, no significant association between stress and duration-based overload registered by EMG was found. The finding of an association between EMA-reported overload and stress may indicate that muscle overload in the presence of TMD pain is not to the same extent linked to a single psychosocial factor such as stress but rather maintained by deeper robust multifactorial mechanisms that have developed over time, whether these are psychosocial (e.g., stress, anxiety, and depression)^58^ or neurobehavioral mechanisms. This interpretation is in line with findings from fibromyalgia research where self-perceived muscle tension correlates more with anxiety-related personality traits than with EMG-recorded muscle activity, suggesting that self-reported tension in chronic pain conditions may reflect emotional or cognitive states rather than physiological muscle hyperactivity^59^.

**Fig. 6:**
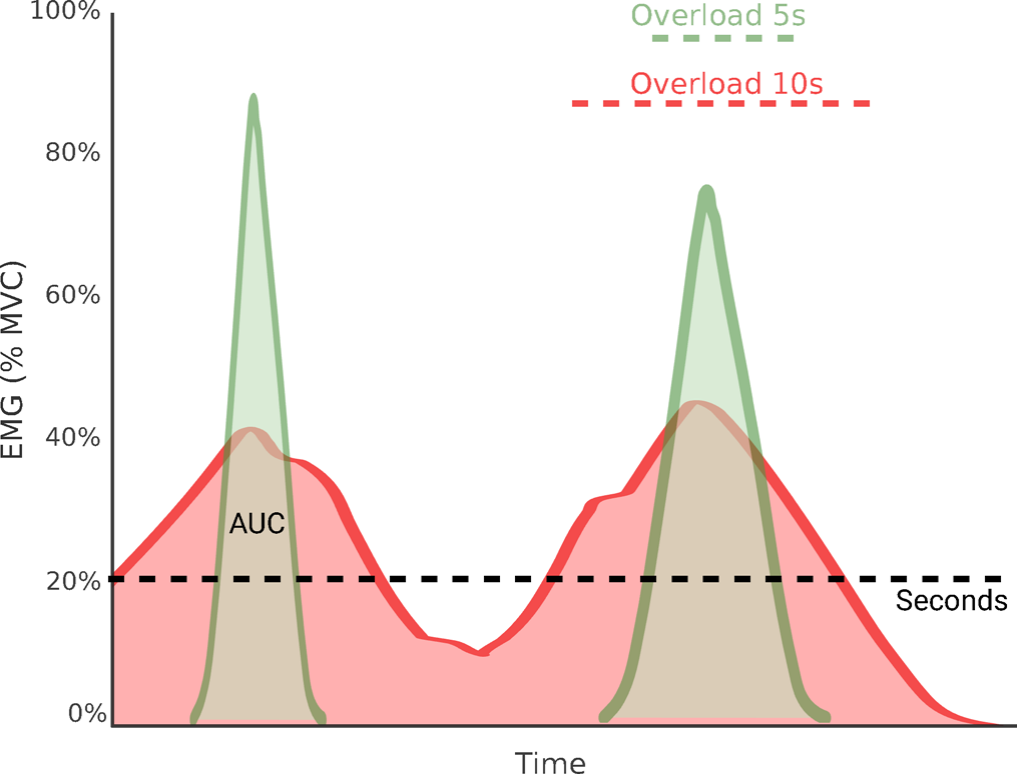
Schematic illustration of electromyography (EMG) activity as percentage of maximum voluntary contraction (MVC) displayed as area under curve (AUC) in individuals with (red) and without (green) pain. Note that despite variations in duration of overload (≥ 20% MVC) for green and red curves (5 vs. 10 s) both exhibit comparable AUC overload values.

Our results showed that in participants without TMD pain, self-reported awake bruxism assessed by EMA was significantly associated with overload duration but not with intensity (AUC). When examining the intensity ranges, the No TMD pain group spent more time in higher MVC levels (60–79%), whereas the TMD group spent more time in lower intensity ranges (20–39%). This may indicate that No TMD pain individuals experience brief but high-level muscle activations, whereas TMD participants are exposed to longer-lasting, low-level overload (Fig. 6). Such differences may reflect different physiological mechanisms or stages in the pain process, where AUC may relate more to acute load risk and duration of overload to long-term load and pain maintenance^6,24^.

The findings of higher levels of both perceived stress and self-reported overload in women compared to men reflect epidemiological patterns in chronic pain conditions, where women commonly exhibit higher pain prevalence and greater psychosocial burden^60^. Furthermore, it has been suggested that psychosocial factors, such as stress, may interact with biological risk factors, such as musculoskeletal overload, to influence the development and maintenance of pain. It is plausible that these dynamics contributed to the elevated self-reported overload in women in our sample, emphasizing the importance of considering gender and psychosocial factors in both the assessment and management of musculoskeletal overload and related pain conditions.

## Strengths and limitations

Our findings support the use of EMA as a meaningful measure of jaw muscle overload that aligns well with objective physiological data such as sEMG. The significant association between EMA-reported awake bruxism and EMG-recorded muscle activity suggests that individuals can reliably self-monitor these behaviours in real time. A possible limitation is that sEMG was recorded only for Day 1, whereas EMA was conducted over three days. The reason for extending the EMA recording over two additional days was to capture possible variation in self-reported non-functional behavior. It was not deemed feasible in the current study to extend sEMG recordings over multiple days, however, future studies may take the day-to-day variability also of sEMG recordings during wakefulness into account.

The 20% MVC threshold to define muscle overload is supported by prior research^37,38,40,41^ and offers a conservative estimate for low-level sustained activity. However, it is possible that a lower threshold could have yielded different results, particularly in detecting more subtle variations in muscle activity. Nevertheless, our finding of more pronounced differences between participants with and without pain in the 20–39% MVC range, suggest that the 20% threshold may indeed capture clinically meaningful differences. EMG was recorded only from the left masseter, which may be considered a limitation. However, prior research indicates a high degree of bilateral symmetry in masseter activity under comparable conditions^61,62^ suggesting that unilateral recordings can provide a reliable reflection of overall masseter activity also considering their synergistic action.

Although efforts were made to minimize the impact from the experimental setup, it is still possible that wearing the EMG device or receiving the EMA prompts may have affected the normal jaw behaviour. Participants might have reduced non-functional jaw activities consciously or subconsciously in response to being observed or prompted. However, the overall levels of self-reported muscle activity when wearing the EMG device were in line with previous EMA studies^42^, suggesting that this possible source of error has not impacted our overall findings.

Taken together, understanding how musculoskeletal overload can contribute to pain requires tools that can capture both behaviour and physiology in real-life contexts. By combining EMA and sEMG, the present study offers a novel approach to simultaneously assess perceived behaviour and muscle activity in the jaw system in a natural environment. Given that muscle overload varies substantially between individuals, situations, and muscle groups—with no universal cut-offs currently established—this integrated method allows for a more individualized and ecologically valid assessment of overload-related behaviour. Because patients are often unaware of low-level jaw-muscle overload, EMA can serve as a clinical tool to document such behaviours and increase awareness. Such a strategy could support targeted discussions on behavioural change and self-management.

## Conclusion

The results suggest that combining EMA and sEMG provides a valid assessment of musculoskeletal overload, capturing both perceptual and physiological dimensions that may provide new insights into possible mechanisms. Given that overload is a risk factor for musculoskeletal pain, the use of EMA to capture real-time behavioural data that is related to objective physiological activity can identify pain-related risk behaviours and thereby support tailored patient-centred interventions.

## Data Availability

The data supporting the findings of this study are available from the corresponding author upon reasonable request. Due to ethical considerations, access to the data is subject to restrictions.
